# Supplementary material for: A core genome approach that enables prospective and dynamic monitoring of infectious outbreaks
Source: Sci Rep. 2019 May 24;9:7808. doi: 10.1038/s41598-019-44189-0 (PMC6534532; doi:10.1038/s41598-019-44189-0)
Supplement: Supplementary file 1 — Supplementary Figures [file 41598_2019_44189_MOESM1_ESM.pdf]

A core genome approach that enables prospective and dynamic monitoring of infectious outbreaks

Helen van Aggelen,[1,*] Raivo Kolde,[1] Hareesh Chamarthi,[1] Joshua Loving,[1] Yu Fan,[1] John T. Fallon, III,[2,3] Weihua Huang,[2] Guiqing Wang,[3] Mary M. Fortunato-Habib,[4] Juan J. Carmona,[4] and Brian D. Gross[4]

[1]Philips Research North America (PRNA), Cambridge, MA, USA; [2]Department of Pathology, New York Medical College (NYMC), Valhalla, New York, USA; [3]Department of Pathology and Clinical Laboratories, Westchester Medical Center (WMC), Valhalla, New York, USA; [4]Philips Healthcare, Genomics for Infectious Disease (G4ID) Unit, Cambridge, MA, USA

**corresponding author: helen.van.aggelen@philips.com*

Supplementary material

	Conserved genes	Conserved sequences
S. aureus	776,385 (28%)	1,843,071 (65%)
E. faecium	530,487 (11%)	994,708 (37%)
K. pneumoniae	1,578,252 (24%)	3,574,338 (67%)

Table S1: Number of nucleotides and the corresponding fraction of the reference genome included in the conserved-gene and conserved-sequence core genomes.

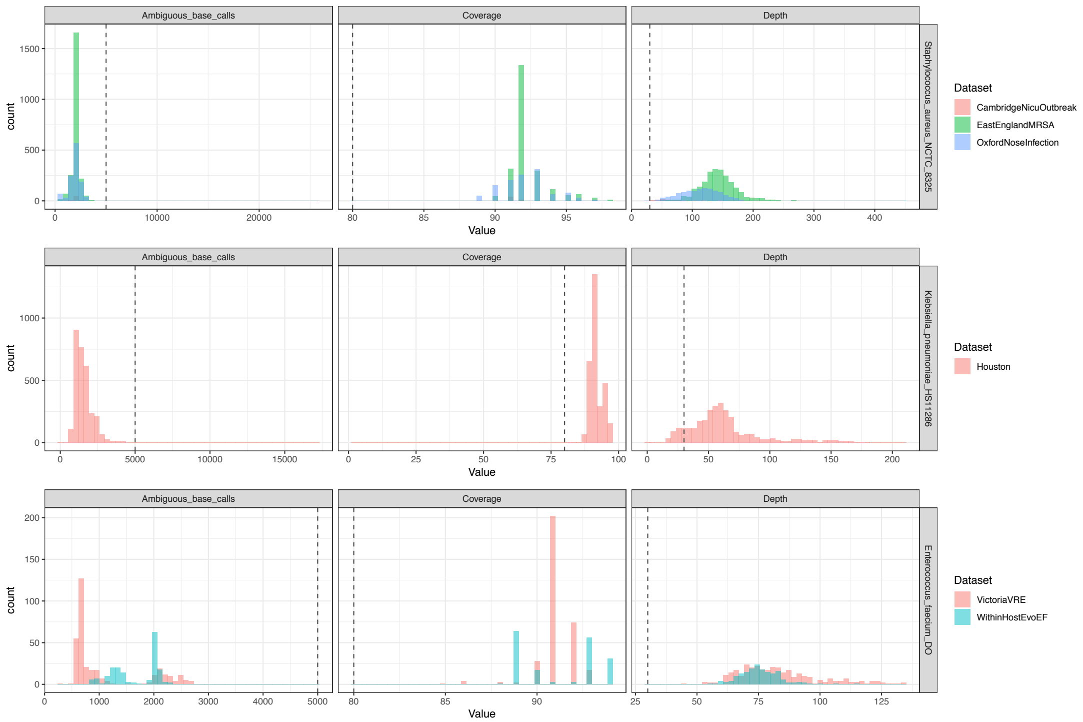

Figure S1: Quality metrics per species (S. aureus first row, K. pneumoniae second row, E. faecium third row) for aligned data and quality thresholds used to include samples in this work.

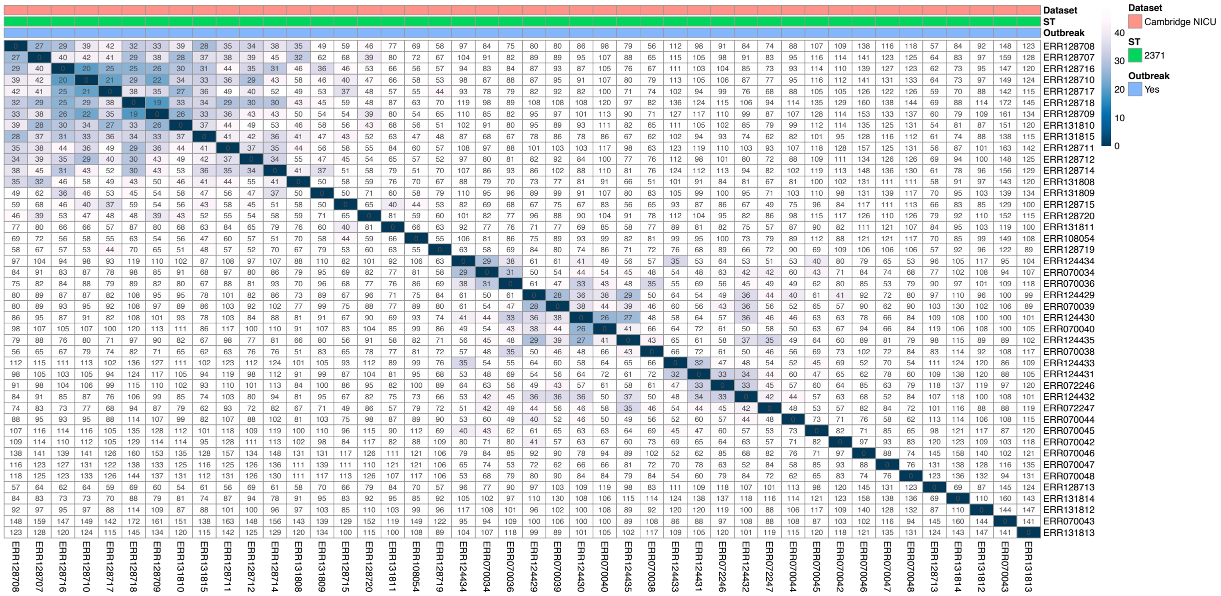

Figure S2: Conserved-gene SNV distances for the S. aureus outbreak cluster (threshold at 49 SNV's). Color band shows the origin and sequence type of samples, and whether they were labeled as part of the outbreak in the outbreak study.

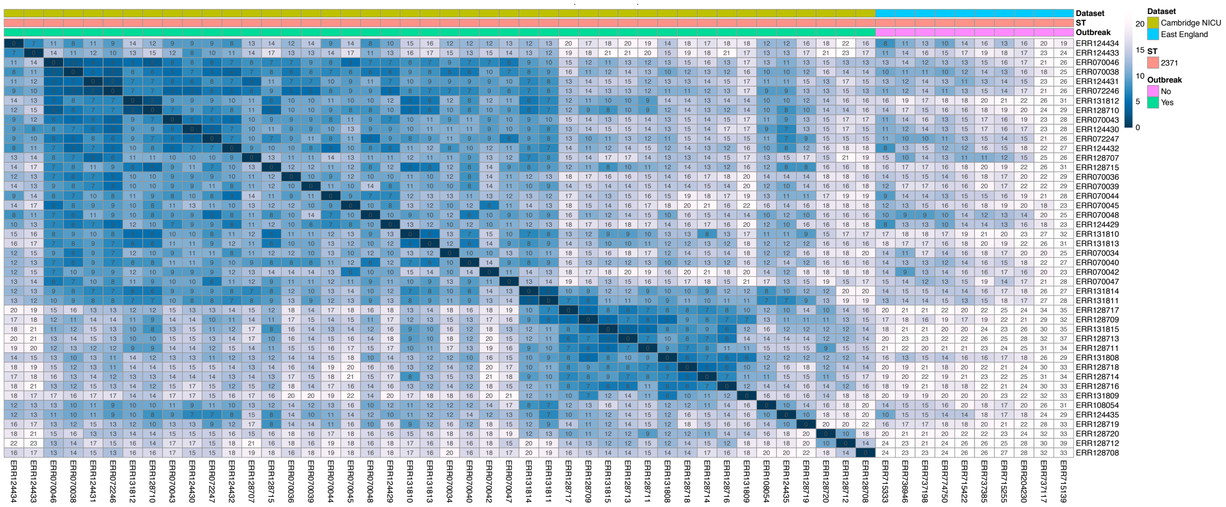

Figure S3: Conserved-sequence SNV distances for the S. aureus outbreak cluster (threshold at 22 SNV's). Color band shows the origin and sequence type of samples, and whether they were labeled as part of the outbreak in the outbreak study (note that the East England samples added to the cluster may be genomically similar but are not labeled as part of the outbreak).

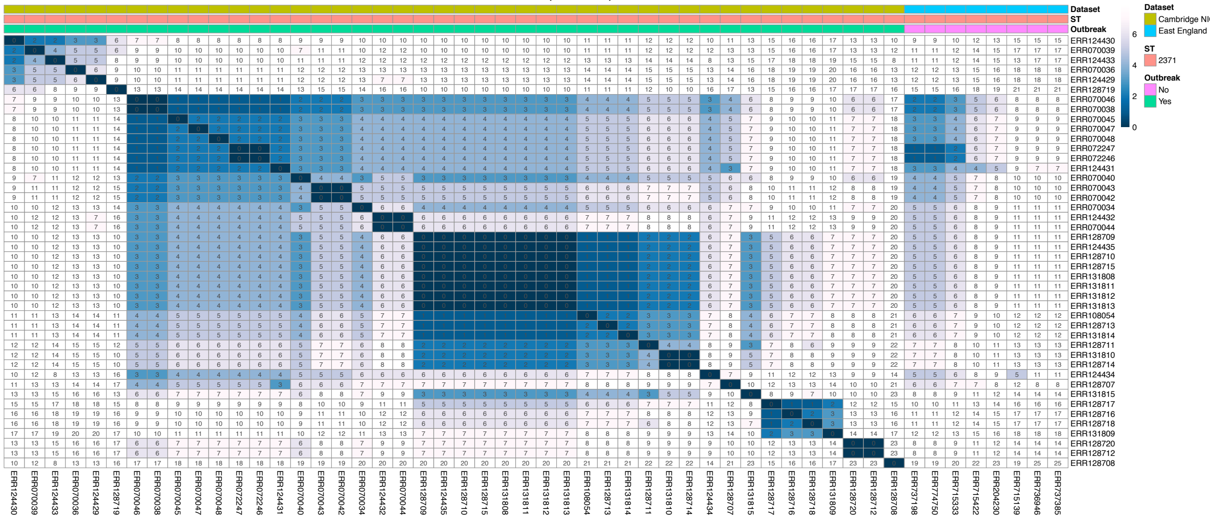

Figure S4: Intersection core genome SNV distances for the S. aureus outbreak cluster (threshold at 7 SNV's). Color band shows the origin and sequence type of samples, and whether they were labeled as part of the outbreak in the outbreak study (note that the East England samples added to the cluster may be genomically similar but are not labeled as part of the outbreak).

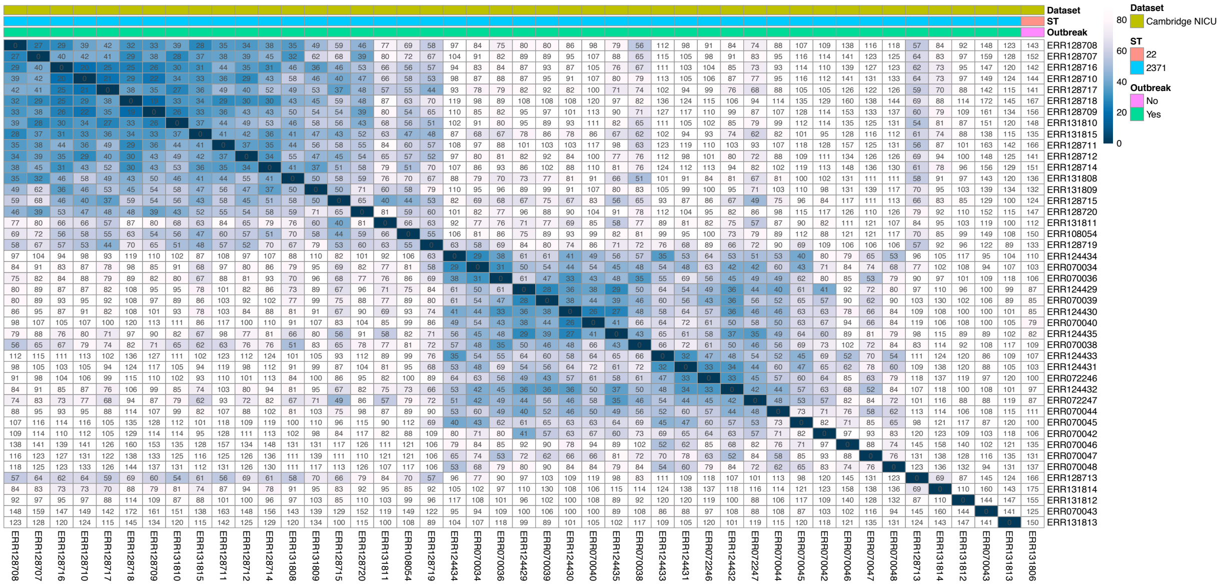

Figure S5: Conserved-gene SNV distances for the S. aureus outbreak cluster (threshold at 89 SNV's). At a threshold of 89 SNV's, all 44 Cambridge outbreak samples are confirmed, although some samples are barely connected. At this threshold, one of the samples confirmed to be unrelated to the outbreak – and of different sequence type – is also included.
